# Endothelin Receptor Autoantibodies as Emerging Biomarkers and Therapeutic Targets in the Cardiovascular Complications of Lupus

**DOI:** 10.21203/rs.3.rs-6465543/v1

**Published:** 2025-04-23

**Authors:** Justin Van Beusecum, Marice McCrorey, Helen Butler, Ryan Lacey, Marharyta Semenikhina, C. Colvert, Kennedy Hawkins, Oleg Palygin, Adviye Ergul, Melissa Cunningham, Jim Oates

**Affiliations:** Medical University of South Carolina and Ralph H. Johnson VAHS; Medical University of South Carolina; Medical University of South Carolina; Medical University of South Carolina; Medical University of South Carolina; Medical University of South Carolina; Medical University of South Carolina and Ralph H. Johnson VAHS; Medical University of South Carolina; Medical University of South Carolina and Ralph H. Johnson VAHS; Medical University of South Carolina; Medical University of South Carolina

## Abstract

Patients with systemic lupus erythematosus (SLE) are at increased risk of hypertension (HTN) and cardiovascular disease, yet the immunologic drivers of this endothelial and vascular dysfunction remain incompletely understood. Here, we investigate whether autoantibodies targeting endothelin receptors are associated with an SLE diagnosis, elevated blood pressure, and endothelial activation. In two independent clinical cohorts (n = 214), we quantified anti-endothelin receptor A (ET_A_R) and endothelin receptor B (ET_B_R) autoantibodies and additionally quantified soluble vascular adhesion molecule-1 (sVCAM-1) and intracellular adhesion molecule-1 (sICAM-1) as markers of endothelial activation. In both independent cohorts, we report significantly elevated anti-ET_A_R autoantibodies, anti-ET_B_R autoantibodies, and sVCAM-1 in individuals with SLE compared to controls. Anti-ET_A_R autoantibodies, Anti-ET_B_R autoantibodies, and sVCAM-1 levels were significantly increased in SLE subjects independent of HTN status. Anti-ET_A_R autoantibodies correlated positively with systolic and diastolic blood pressure, sVCAM-1, sICAM-1, and anti-ET_B_R autoantibodies. These findings identify a novel immunological signature of endothelial dysfunction in SLE and implicate anti-endothelin receptor autoantibodies as potential biomarkers and therapeutic targets in SLE and its associated HTN.

## INTRODUCTION

Systemic Lupus Erythematosus (SLE) is a complex, chronic autoimmune disease with a striking sex bias, affecting women up to nine times more frequently than men, particularly during reproductive years^1,2^. This disproportionate burden on women underscores a critical need to investigate disease mechanisms, complications, and therapeutic opportunities to improve clinical care for women. Among the most severe and underrecognized complications of SLE in women is cardiovascular disease (CVD), which contributes significantly to premature morbidity and mortality worldwide^3^. Women with an SLE diagnosis are at increased risk for stroke, myocardial infarction, and peripheral artery disease, often decades earlier than their female counterparts in the general population^4–6^.

Hypertension (HTN), a primary modifiable risk factor for CVD complications^7–9^, is significantly more prevalent in women with SLE relative to non-SLE controls despite comparable preventative strategies and therapeutic management methods^10,11^. These clinical observations suggest that traditional risk factors, mechanisms, and therapies alone do not fully account for the high incidence of HTN and vascular complications in women with SLE. Immune-mediated mechanisms, including autoantibody-driven endothelial dysfunction, may represent a novel contributor to the cardiovascular complications that occur in patients with SLE. However, the molecular mediators linking immune dysregulation, autoantibodies, HTN, and subsequent CVD events, including endothelial cell activation in SLE patients, remain incompletely defined.

The endothelin (ET) signaling axis, comprising of the vasoconstricting peptide endothelin-1 (ET-1) and its cognate G-protein coupled receptors, endothelin receptor A (ET_A_R) and endothelin receptor B (ET_B_R), have been implicated in both autoimmunity^12,13^ and HTN^14–17^. Dysregulated ET-1, ET_A_R, and ET_B_R signaling have been observed in murine models of SLE, and pharmacological inhibition with endothelin receptor antagonists (ERAs) has proven beneficial^18,19^. Clinically, ERAs have been employed in the management of pulmonary arterial hypertension (PAH)^20^, resistant hypertension^21^, and glomerular disorders such as IgA nephropathy^22^.

Recent work has identified autoantibodies targeting endothelial receptors as potential mediators of vascular inflammation and immune cell recruitment in the pathogenesis of autoimmune conditions^23–25^. Anti-endothelin receptor A (anti-ET_A_R) autoantibodies isolated from patients with systemic sclerosis (SSc)-associated pulmonary arterial hypertension (PAH) have been demonstrated to induce pathological endothelial cell calcium fluxes in pulmonary arteries^25^. In SLE, however, the presence of anti-ET_A_R autoantibodies and anti-endothelin receptor B (anti-ET_B_R) autoantibodies remain largely explored. Two prior studies have shown that SLE-derived autoantibodies, anti-Factor Xa and anti-ET_A_R autoantibodies, can induce calcium fluxes and inflammatory responses in endothelial and vascular smooth muscle cells^24,26^. However, whether anti-ET_A_R and anti-ET_B_R autoantibodies are linked to SLE-associated HTN has not been determined.

In the present study, we report the first evidence of the presence and clinical significance of anti-ET_A_R and anti-ET_B_R autoantibodies in SLE human subjects with and without systemic HTN across two independent clinical cohorts. We identify strong associations between these novel autoantibodies and hypertensive status and correlations with endothelial activation and dysfunction biomarkers. These findings suggest that autoantibody-mediated targeting of endothelin receptors may describe a novel mechanism contributing to vascular complications in SLE patients. Our study advances the understanding of sex-specific cardiovascular risk in autoimmunity. Moreover, it highlights a potential path toward biomarker-guided risk stratification and therapeutic intervention in patients with autoimmune diseases, specifically an SLE diagnosis.

## PATIENTS AND METHODS

### Data availability:

The data that support the findings will be available upon request.

### Human Subjects

Female non-SLE (n=20) and SLE (n=17) female human subjects were recruited between the ages of 23 and 64. Clinical and demographic characteristics are shown in Table 1.Current therapies for all human subjects are shown in Table 2. Female human subjects were considered to have SLE if they had a new or existing diagnosis of SLE based on either the 2021 SLE International Collaborating Clinics (SLICC) criteria^27^ or the 2019 American College of Rheumatology/European League Against Rheumatism (ACR/EULAR) criteria for SLE^28^. Female human subjects were considered hypertensive if they had a systolic blood pressure >130 mmHg or a diastolic blood pressure >85 mmHg or had a diagnosis of HTN and were currently treated with antihypertensive agents. Blood pressure was obtained as the average of three determinations using an automatic device after 15 minutes of rest with the human subject sitting upright and their back supported and feet on the floor. Hypertensive human subjects were defined as those with systolic blood pressures >130 mmHg, while normotensive participants had blood pressures <130 mmHg. Race as a social construct was self-defined in this study.

Exclusion criteria included the following: (1) diabetes mellitus, Type I or II, (2) active malignancy, (3) severe psychiatric disorders, (4) HIV/AIDS, or (5) pregnancy.

In a second independent clinical cohort, plasma from non-SLE non-HTN healthy controls (n=45), non-SLE HTN (n=36), SLE non-HTN (n=47), and SLE-HTN (n=49) female and male human subjects between the ages of 18 and 81 were obtained from the Medical University of South Carolina Core Center for Clinical Research (CCCR) Biorepository. Clinical and demographic characteristics are shown in Table 3. Human subjects were considered to have SLE if they had a new or existing diagnosis of SLE based on either the 2021 SLE International Collaborating Clinics (SLICC) criteria^27^ or the 2019 American College of Rheumatology/European League Against Rheumatism (ACR/EULAR) criteria for SLE^28^. Human subjects were considered hypertensive if they had a systolic blood pressure >130 mmHg or a diastolic blood pressure > 85 mmHg or had a diagnosis of HTN and were currently treated with antihypertensive agents at the time of blood draw.

Exclusion criteria included the following: (1) diabetes mellitus, Type I or II, (2) active malignancy, (3) severe psychiatric disorders, (4) HIV/AIDS, or (5) pregnancy. The protocol (PRO# 122811) was approved by the Medical University of South Carolina Institutional Review Board and conformed to the standards of the US Federal Policy for the protection of Human Subjects and in accordance with the World Medical Association Declaration of Helsinki.

### Measurement of plasma endothelial activation markers and endothelin receptor autoantibodies:

Human subject plasma was aliquoted and stored at −80°C until further analysis. Plasma was used for the measurement of anti-ET_A_R autoantibodies using EIA for Quantitative Determination of anti-Endothelin Receptor A (ETA)-Antibodies ELISA kit (CellTrend; Cat #12100), and anti-ET_B_R autoantibodies using EIA for Quantitative Determination of anti-Endothelin Receptor B (ETB)-Antibodies (CellTrend; Cat #13300), sVCAM-1 using Human VCAM-1/CD106 Quantikine ELISA kit (R&D Systems; DVC00), and sICAM-1 using Human ICAM-1/CD54 Allele-specific Quantikine ELISA kit (R&D Systems; DCD540), as per manufacturer’s instructions (**Supplemental Table 1**).

### Statistical Analysis:

All samples were given a random identifier for analysis of human subject plasma so that the experiment and analysis were performed blinded. GraphPad Prism Version 10 was used to perform statistical analyses. The D-Agostino and Pearson test was performed with an a set to 0.05 to test values for normality. A Grubbs test was employed with an a set to 0.05 to ensure values were not outliers. All data sets in the tables are expressed as mean ± SD, while all data sets in the figures are expressed as mean ± SEM. were expressed as a mean ± SEM. As the hypothesis was that anti-ET_A_R autoantibodies, anti-ET_B_R autoantibodies, and endothelial activation biomarkers levels would be elevated in human subjects with SLE, we utilized a one-tailed Mann-Whitney test for studies comparing the effect of SLE diagnosis. As all the data was non-parametric, a non-parametric Kruskal-Wallis with Dunn’s multiple comparisons was employed for studies comparing the effect of SLE or HTN diagnosis on plasma anti-ET_A_R autoantibodies, anti-ET_B_R autoantibodies, and sVCAM-1. Female and male subjects were analyzed independently utilizing a non-parametric Kruskal-Wallis test with Dunn’s multiple comparisons for plasma anti-ET_A_R autoantibodies, anti-ET_B_R autoantibodies, and sVCAM-1. To determine the associations between anti-ET_A_R autoantibodies and sVCAM-1 and anti-ET_B_R autoantibodies and sVCAM-1 in the pilot cohort, a non-parametric Spearman r correlation was employed. A non-parametric Spearman r correlation matrix was used to summarize the strength and directionality between the multiple variables assessed within this study (plasma anti-ET_A_R autoantibodies, plasma anti-ET_B_R autoantibodies, plasma sVCAM-1, plasma sICAM-1, systolic blood pressure, diastolic blood pressure, and pulse pressure). The same variables were standardized and evaluated by a correlative principal component analysis (PCA) using RStudio Version 2024.12.1+563. P values were reported in the figures and were considered significant when <0.05.

## RESULTS

### Autoantibodies targeting endothelin receptors are elevated in SLE

**Figure 1:**
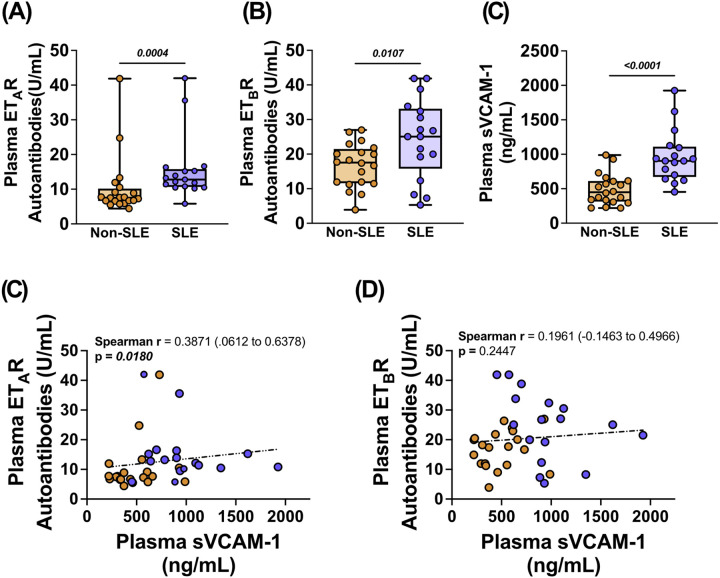
Anti-endothelin receptor autoantibodies are elevated in a pilot cohort of female SLE subjects and associated with endothelial activation. Plasma from female subjects with or without SLE was analyzed for plasma anti-endothelin receptor A (anti-ET_A_R) autoantibodies and anti-endothelin receptor B (anti-ET_B_R)autoantibodies titers. Mean data for plasma titers of **(A)** anti-ET_A_R autoantibodies, **(B)** anti-ET_B_R autoantibodies, and **(C)** soluble vascular cell adhesion molecule-1 (sVCAM-1) in non-SLE (n=20) and SLE subjects(n=17). Correlation analysis of plasma titers for **(D)** anti-ET_A_R autoantibodies and sVCAM-1, and **(E)** anti-ET_B_R autoantibodies and sVCAM-1 in all subjects (n=37). All data is expressed as mean ± SEM. Data in panels (**A-C**) were analyzed using a one-tailed Mann-Whitney test. Data in panels (**D-E**) were analyzed using a Spearman r correlation. P values and confidence intervals are indicated within each respective graph.

To initially determine whether autoantibodies targeting endothelin receptors are associated with SLE, we quantified circulating levels of anti-ET_A_R and anti-ET_B_R autoantibodies in plasma from female non-SLE and SLE human subjects. Both anti-ET_A_R and anti-ET_B_R autoantibodies were significantly elevated in SLE subjects compared to non-SLE controls (p = 0.0004 and p = 0.0107, respectively; Fig. 1A and B; **Supplemental Tables 2 and 3**).

Endothelial activation and vascular inflammation are hallmark features of cardiovascular dysfunction and are commonly assessed through soluble adhesion molecules, including vascular cell adhesion molecule-1 (sVCAM-1)^29^. To investigate whether SLE influences endothelial activation, we quantified circulating levels of sVCAM-1 in SLE and non-SLE subjects. Plasma sVCAM-1 levels were significantly elevated in female SLE subjects compared to non-SLE controls (p < 0.0001; Fig. 1C; **Supplemental Table 4**). To explore potential associations between autoantibody titers and endothelial activation, we examined correlations between anti-ET_A_R and anti-ET_B_R autoantibodies with plasma sVCAM-1. We found positive associations between plasma anti-ET_A_R autoantibodies and sVCAM-1 (Spearman r = 0.3871, p = 0.0180; Fig. 1D; Supplemental Table 5). However, no statistical association was observed between anti-ET_B_R autoantibodies and sVCAM-1 (Spearman r = 0.1962; p = 0.2447; Fig. 1E; **Supplemental Table 6**), suggesting a more prominent role of anti-ET_A_R autoantibodies in endothelial cell activation.

### Anti-ET_A_R and anti-ET_B_R autoantibodies are elevated in SLE with and without HTN

**Figure 2:**
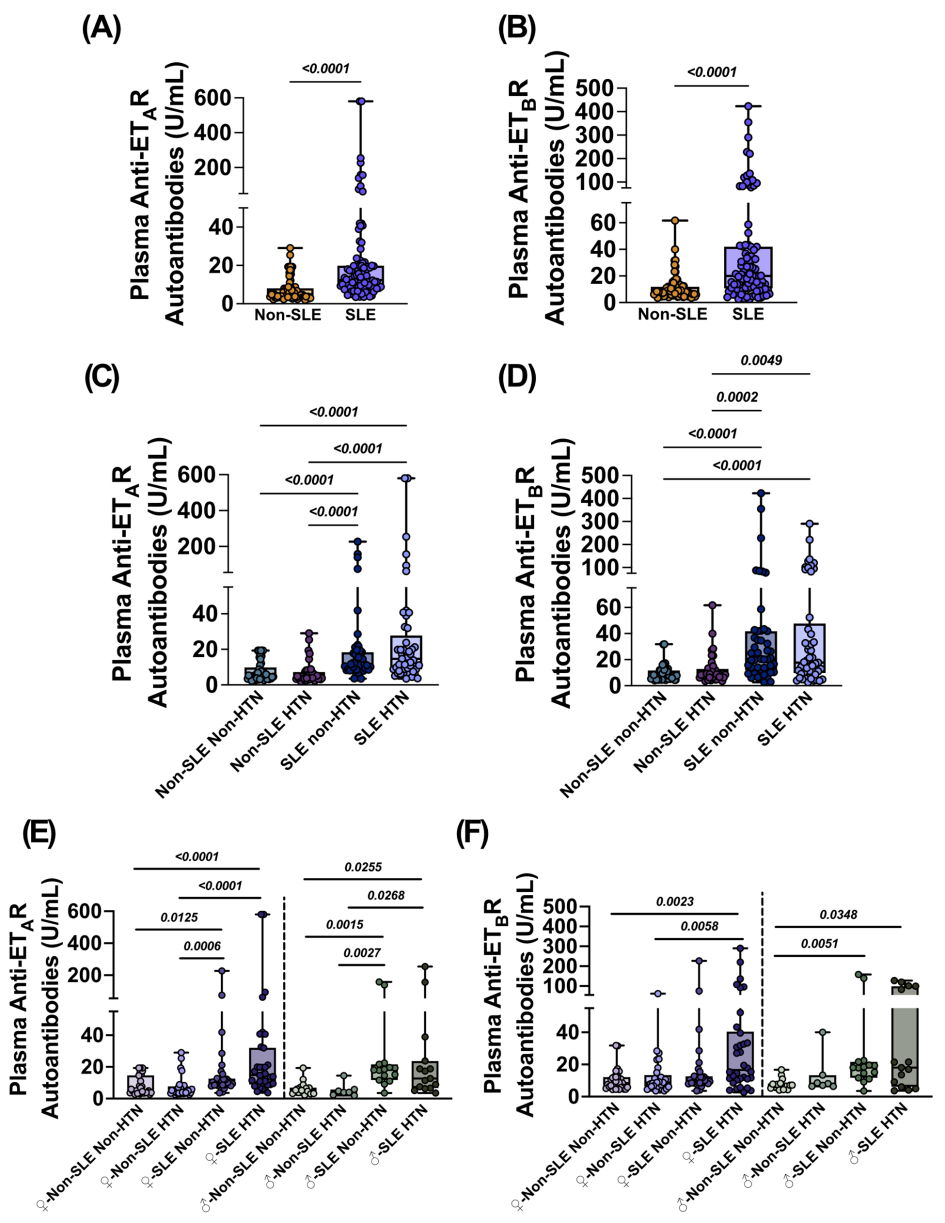
Anti-Endothelin Receptor Autoantibodies are Elevated in SLE Associated Hypertension. Plasma from female and male subjects with or without SLE and/or hypertension (HTN) was analyzed for anti-endothelin receptor A (anti-ET_A_R) and anti-endothelin receptor B (anti-ET_B_R) autoantibody titers. Mean data for plasma titers of **(A)** anti-ET_A_R and **(B)** anti-ET_B_R autoantibodies in non-SLE (n=81) and SLE subjects(n=96). Mean data for plasma titers of **(C)** anti-ETAR and **(D)** anti-ET_B_R autoantibodies in non-SLE non-HTN (n=45), non-SLE HTN (n=36), SLE non-HTN (n=47), and SLE HTN (n=49) human subjects. Mean data for plasma titers of **(E)** anti-ET_A_R and **(F)** anti-ET_B_R autoantibodies stratified by sex with male subjects on the left side and female subjects on the right side of their respective panel. All data is expressed as mean ± SEM. Data in panels (**A and B**) were analyzed using a one-tailed Mann-Whitney U test. Data in panels (**C-F**) were analyzed using a non-parametric Kruskal-Wallis test with Dunn’s multiple comparisons test. Female and male subjects were analyzed statistically independently of each other. P values are indicated within each respective graph.

To validate these findings, we analyzed anti-ET_A_R and anti-ET_B_R autoantibodies in a larger clinical cohort stratified by SLE and HTN status. Circulating anti-ET_A_R and anti-ET_B_R autoantibodies were significantly elevated in SLE subjects compared to non-SLE controls (p < 0.0001 for both; Fig. 2A and B; **Supplemental Tables 7 and 8**). When stratified by HTN diagnosis, anti-ET_A_R autoantibodies were elevated in subjects with an SLE diagnosis irrespective of HTN status, to both non-SLE non-HTN (p < 0.0001 for both) and non-SLE HTN (p < 0.0001 for both) groups (Fig. 2C **and Supplemental Table 9**). Similarly, anti-ET_B_R autoantibodies were elevated in SLE subjects regardless of HTN status, with comparable levels between SLE-HTN and SLE non-HTN groups (Fig. 2D **and Supplementary Table 10**).

Sex-stratified analyses revealed distinct patterns in anti-ET_A_R autoantibody elevation. In females, anti-ET_A_R autoantibodies were significantly elevated in both SLE non-HTN and SLE-HTN subjects compared to non-SLE HTN (p = 0.0125 and p = 0.0006, respectively) and non-SLE non-HTN subjects (p < 0.0001 for both comparisons; Fig. 2E and **Supplemental Table 11**). Similarly, male SLE non-HTN and SLE-HTN subjects exhibited significantly higher anti-ET_A_R autoantibody levels relative to non-SLE HTN (p = 0.0015 and p = 0.0268, respectively) and non-SLE non-HTN groups (p = 0.0027 and p = 0.0255; Fig. 2E **and Supplemental Table 12**). Notably, the magnitude of anti-ET_A_R autoantibody elevation was more pronounced in female SLE subjects compared to their respective control groups, indicating a potential sex bias in anti-ET_A_R autoantibody-associated endothelial activation and dysfunction.

In females, circulating levels of anti-ET_B_R autoantibodies were significantly elevated in the SLE-HTN group compared to both non-SLE non-HTN (p = 0.0023) and non-SLE HTN (p = 0.0058) groups (Fig. 2F **and Supplemental Table 13**). In contrast, male SLE subjects, regardless of hypertensive status, exhibited increased anti-ET_B_R autoantibody levels only in comparison to non-SLE non-HTN controls (SLE non-HTN: p = 0.0051; SLE-HTN: p = 0.00348; Fig. 2F **and Supplemental Table 14**). These findings suggest a sex-specific pattern in anti-ET_B_R autoantibody elevation, implicating a potential sexual dichotomy in their association with SLE-related HTN.

### Sex-specific elevation of endothelial activation marker sVCAM-1 in female SLE patients with comorbid HTN

**Figure 3:**
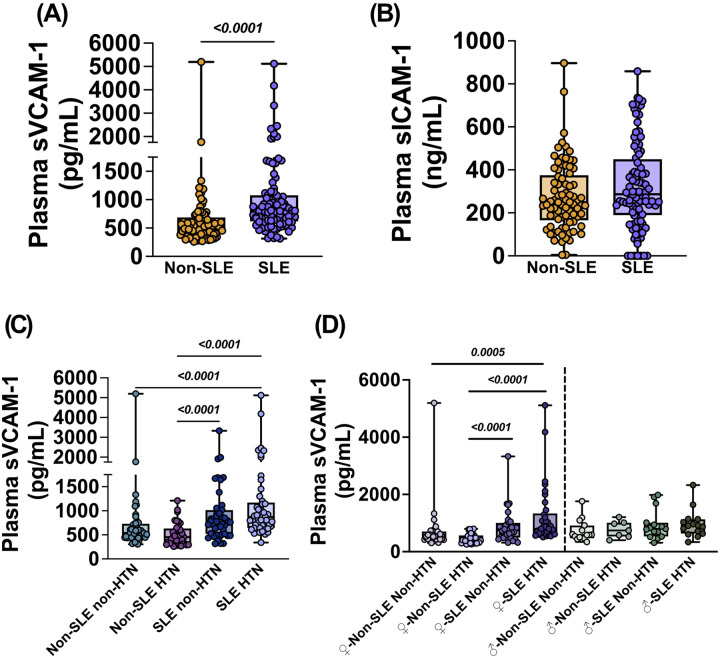
Endothelial activation biomarkers are elevated in SLE-associated HTN. Plasma from female and male subjects with or without SLE and/or hypertension (HTN) was analyzed for plasma soluble vascular adhesion molecule-1 (sVCAM-1) and soluble intracellular adhesion molecule-1 (sICAM-1) concentrations. Mean data for plasma concentrations of **(A)** sVCAM-1 and **(B)** sICAM-1 in non-SLE (n=81) and SLE subjects(n=96). **(C)** Mean data for plasma concentrations of sVCAM-1 in non-SLE non-HTN (n=45), non-SLE HTN (n=36), SLE non-HTN (n=47), and SLE HTN (n=49) human subjects. **(D)** Mean data for plasma concentrations of sVCAM-1 stratified by sex with male subjects on the left side and female subjects on the right side of their respective panel. All data is expressed as mean ± SEM. Data in panels (**A and B**) were analyzed using a one-tailed Mann-Whitney U test. Data in panels (**C and D**) were analyzed using a non-parametric Kruskal-Wallis test with Dunn’s multiple comparisons test. Female and male subjects were analyzed statistically independently of each other. P values are indicated within each respective graph.

We next examined how SLE and HTN interact to influence endothelial and vascular biomarkers. We found sVCAM-1 was significantly elevated in SLE compared to non-SLE subjects (p < 0.0001; Fig. 3A; **Supplemental Table 15**), while sICAM-1 levels remained unchanged (Fig. 3B; **Supplemental Table 16**). Stratification by HTN status revealed that sVCAM-1 was profoundly elevated in SLE-HTN subjects relative to non-SLE HTN (p < 0.0001) and non-SLE HTN (p < 0.0001) individuals. In contrast, SLE non-HTN was elevated compared to non-SLE non-HTN controls (p < 0.0001; Fig. 3C **and Supplementary Table 17**).

Further stratification by sex identified a striking effect in female patients. sVCAM-1 levels significantly increased in female SLE-HTN subjects compared to non-SLE HTN (p < 0.0001) and non-SLE non-HTN p = 0.0005), controls, while SLE non-HTN subjects were significantly increased compared to non-SLE non-HTN individuals (p < 0.0001; Fig. 3D **and Supplementary Table 18**). In stark contrast, no significant changes in sVCAM-1 were observed among male subjects across the diagnosis groups. These findings suggest that endothelial activation, as clinically reflected by sVCAM-1, is preferentially elevated in females with SLE and coexisting HTN.

### Anti-endothelin receptor autoantibodies are associated with blood pressure and endothelial dysfunction in SLE

**Figure 4:**
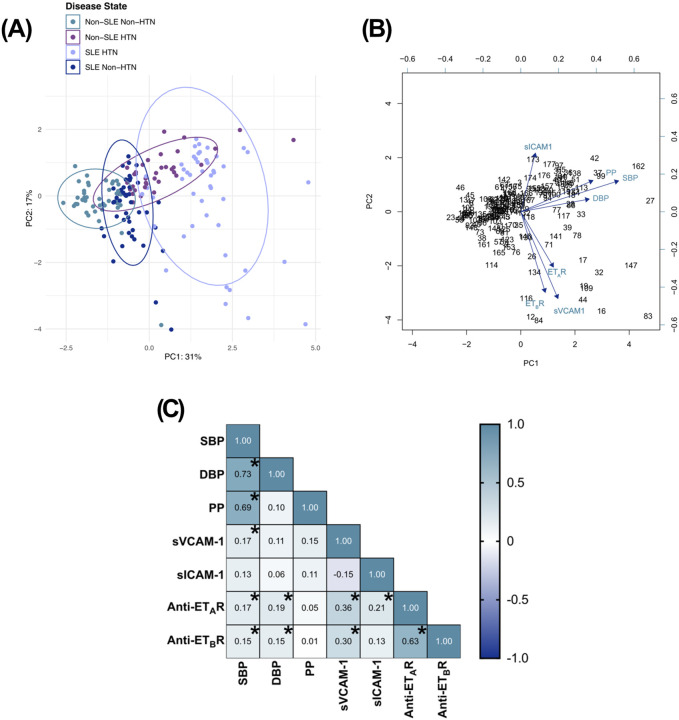
Anti-endothelin receptor autoantibodies correlate with endothelial activation and blood pressure in SLE. **(A)** A principal component analysis (PCA) was performed with the parameters assessed, including systolic blood pressure (SBP), diastolic blood pressure (DBP), calculated pulse pressure (PP), sICAM-1, sVCAM-1, anti-anti-endothelin receptor A (anti-ET_A_R) autoantibodies, and (anti-ET_B_R) autoantibodies from all 177 human subjects. **(B)** The loadings plot from the PCA demonstrates the driving factors that skew human subjects towards an SLE and/or hypertension diagnosis. **(C)** A correlation matrix is shown to summarize the strength and directionality of the relationships of all assessed parameters to each other. Significant correlations are expressed with an asterisk. A correlative PCA-evaluated standardized data was used to analyze panels **A and B.** In panel **C,** Spearman’s rank correlation coefficients with α = 0.05 were used for correlation matrix calculations.

To evaluate how the multiple parameters assessed contribute to variance between diagnostic groups, we performed a principal component analysis (PCA) incorporating plasma levels of anti-ET_A_R autoantibodies, anti-ET_B_R autoantibodies, sVCAM-1, sICAM-1, and blood pressure measurements. PCA revealed a distinct cluster of SLE-HTN subjects separated from the other groups (Fig. 4A **and Supplemental Table 20**). Principal component 1 (PC1), which accounted for 31% of the variance, was driven by systolic blood pressure (0.647), pulse pressure (0.478), and diastolic blood pressure (0.454), while principal component 2 (PC2; 17% variance) showed high loadings for sVCAM-1 (−0.576) and anti-ET_B_R autoantibodies (−0.536; Fig. 4B **and Supplemental Table 20**).

Correlation matrix analysis confirmed positive associations between anti-ET_A_R autoantibodies and systolic blood pressure (r = 0.169; p = 0.024), diastolic blood pressure (r = 0.190; p = 0.011), sVCAM-1 (r = 0.361; p = < 0.0001), and sICAM-1 (r = 0.207; p = 0.006; Fig. 4C **and Supplemental Table 21**). Anti-ET_B_R autoantibodies were also positively correlated and systolic blood pressure (r = 0.0151; p = 0.045), diastolic blood pressure (r = 0.152; p = 0.044) and sVCAM-1 (r = 0.300; p > 0.0001; Fig. 4C **and Supplemental Table 21**). Lastly, a strong positive correlation was observed between anti-ET_A_R and anti-ET_B_R autoantibodies (r = 0.631; p = < 0.0001; Fig. 4C).

Taken together, these data identify anti-ET_A_R and anti-ET_B_R autoantibodies as novel circulating biomarkers associated with blood pressure and endothelial dysfunction in patients with SLE. Their enrichment in female SLE-HTN subjects suggests a potential mechanistic link contributing to sex-specific cardiovascular risk in the pathogenesis of SLE.

## DISCUSSION

SLE is a heterogeneous and devasting disease associated with a range of cardiovascular complications, including the development of HTN^30,31^. In the present study, we explored the association between the presence of endothelin receptor-specific autoantibodies in SLE and how HTN may contribute to the presence of endothelin receptor-specific autoantibody titers in human subjects. We report elevated anti-ET_A_R and anti-ET_B_R autoantibodies in SLE-associated systemic HTN. Additionally, while there is a higher prevalence of SLE in women relative to men^2,32^, here we demonstrate elevated autoantibodies in both males and females with SLE. Importantly, we highlight the association between anti-ET_A_R and anti-ET_B_R autoantibodies with prominent markers of endothelial activation/vascular inflammation and vascular dysfunction in SLE and SLE-HTN human subjects. Lastly, our analysis further revealed that the combination of anti-ET_A_R and anti-ET_B_R autoantibody titers, sVCAM-1, and blood pressure measurements distinguished SLE and SLE-HTN subjects from their respective controls in a multivariate analysis. These findings provide novel clinical relevance to understanding the role of circulating anti-ET_A_R and anti-ET_B_R autoantibodies in potentiating 1.) vascular inflammation and dysfunction, 2.) differential effects on endothelial cells and vascular smooth muscle cells, and 3.) imparting functional, receptor-specific vascular effects.

Mechanistically, the cognate receptors within the endothelin system (ET_A_R and ET_B_R) are important in maintaining vascular tone and blood pressure homeostasis^33^. The vascular smooth muscle cells (SMCs) predominantly express ET_A_R^34^, resulting in strong calcium-induced SMC contraction and subsequent vessel constriction^35^. ET_B_R, mainly expressed by the vascular endothelium, regulates nitric oxide production and acts juxtaposed to ET_A_R to induce vessel dilation^36^. Dysregulation of ET_A_R and ET_B_R signaling through autoantibody engagement may lead to imbalanced vascular tone, inflammation, and subsequent vascular injury. While the pathogenic role of anti-ET_A_R autoantibodies has been described in systemic sclerosis-associated pulmonary arterial hypertension (SSc-PAH)^24,37–39^, their role in SLE remains largely unexplored. Our findings suggest that similar pro-inflammatory mechanisms may be pathological in SLE and potentially contribute to endothelial and smooth muscle dysfunction.

Endothelial activation is reported in numerous studies to occur secondary to hypertensive insults and vascular dysfunction^40,41^. Of particular interest, we observed elevated sVCAM-1 levels in female SLE subjects with HTN, while sICAM-1 levels remained unchanged. This selective elevation supports a model in which anti-ET_A_R and possibly anti-ET_B_R autoantibodies promote vascular dysfunction and inflammation by upregulating specific adhesion molecules independent of hypertensive injury. Previous reports demonstrating the pro-inflammatory effects of anti-ET_A_R autoantibodies in human endothelial cells may provide evidence of induced endothelial activation and inflammation in SLE due to the presence of elevated anti-ETAR autoantibodies^24,38^. Elevated anti-ET_B_R autoantibodies have been reported in systemic sclerosis (SSc) patients with secondary PAH^25^. However, no studies have described their role in SLE as a potential mediator of endothelial activation and subsequent vascular dysfunction. The findings of elevated anti-ET_B_R autoantibodies in SLE subjects and their positive association with endothelial activation warrant further exploration of the functional effects of anti-ET_B_R autoantibodies on the vascular endothelium. The potential imbalances in endothelin signaling imparted by anti-ET_A_R and anti-ET_B_R autoantibodies may lead to increased end-organ inflammation, subsequent organ damage, and cardiovascular disease development in SLE.

Currently, there are no widely established clinical guidelines for using anti-ET_A_R autoantibodies in clinically diagnosing SLE. However, studies have highlighted its potential as a biomarker in patients diagnosed with SSc-associated PAH^24,37,38^. Furthermore, Avouac et al. demonstrated anti-ET_A_R-AAs titer levels as a biomarker for predicting digital ulcer formation in SSc patients in five-year follow-ups^39^. Our study utilized a multivariant analysis method to determine the contributing factors resulting in SLE subjects’ distinct clustering. The findings of anti-ET_A_R and anti-ET_B_R autoantibodies are driving factors skewing subjects toward an SLE diagnosis and highlight the potential utility of these biomarkers as a biosignature for SLE-associated vascular dysfunction and subsequent inflammation.

Our study has several limitations. All human subjects received stand-of-care treatments for SLE and/or HTN at the time of analysis, which may have modulated biomarker plasma levels. Additionally, we elucidated differences in anti-ET_A_R and anti-ET_B_R autoantibody titer levels within each sex compared to their sex-matched controls, and we were not statistically powered to determine if any sex-specific differences occurred between SLE and SLE HTN females and males. We also did not assess cytokine profiles or type I interferon responses, which are known to contribute to endothelial activation and dysfunction in SLE. Finally, we did not directly assess the functional impact of anti-ET_A_R and anti-ET_B_R autoantibodies on vascular cells (vascular smooth muscle and endothelial cells) in vitro, an important next step in elucidating their mechanistic role in the pathogenesis of SLE. Nevertheless, our data identify plasma anti-ET_A_R and anti-ET_B_R autoantibodies as promising and novel biomarkers of vascular inflammation and dysfunction in patients with SLE. Their profound correlation with endothelial activation markers, specifically sVCAM-1, and their enrichment in SLE-HTN subjects support their potential as contributors to the development of cardiovascular disease in SLE. These findings provide novel avenues for investigating non-canonical endothelin system signaling and for developing targeted therapies that modulate anti-endothelin receptor interactions.

Our study highlights anti-ET_A_R and anti-ET_B_R autoantibodies as potential novel signaling moieties in SLE associated with blood pressure dysregulation, endothelial activation, and distinct vascular biomarker profiles. Anti-ET_A_R and anti-ET_B_R autoantibodies may reveal previously unknown mechanisms of endothelial/vascular dysfunction and inflammation in SLE. These findings underscore the need for translational research bridging autoimmunity and vascular biology to inform new diagnostic and therapeutic strategies in SLE and its associated cardiovascular complications. Future studies to elucidate receptor-specific autoantibody functions, sex-dependent mechanisms, and longitudinal outcomes will be critical to advancing our understanding and management of SLE-associated cardiovascular disease. Altogether, our data support the feasibility of a clinical trial on the antagonism of the ET system and a potential non-canonical ET receptor signaling mechanism in patients with SLE and its associated HTN.

## Figures and Tables

**Table 1:** Clinical Characteristics and Demographics of Pilot Cohort for Plasma Anti-Endothelin Receptor Autoantibody Biomarkers

	Non-SLE	SLE	Overall
N=	20	17	37
Age	44 ± 14	42 ± 14	43 ± 14
Sex (F/M)	20/0	17/0	37/0
Race (W/B/A/O)	(9/10/0/1)	(6/11/0/0)	(15/21/0/1)
Height (cm)	169.9 ± 9.1	163.9 ± 6.6	165.0 ± 8.1
Weight (Kg)	81.2 ± 22.4	75.0 ± 23.8	78.4 ± 22.9
BMI (Kg/m^2^)	29.8 ± 8.3	27.6 ± 7.6	28.8 ± 7.9
Hypertensive (%)	10 (50)	9 (53)	19 (51)
Office SBP (mmHg)	121 ± 14	123 ± 17	122 ± 15
Office DBP (mmHg)	83 ± 9	81 ± 9	82 ± 9
SLEDAI Index	-	1.8 ± 2.4	-

Systemic lupus erythematosus: SLE, Body mass index: BMI, Systolic blood pressure: SBP, Diastolic blood pressure: DBP, Systemic lupus erythematosus disease activity index: SLEDAI. All data are expressed as mean ± SD. Percentages of the population in paratheses when indicated.

**Table 2:** Current Medications for Pilot Cohort Subjects Studied for Anti-Endothelin Receptor Autoantibody Biomarkers.

	Non-SLE	SLE	Overall
N=	20	17	37
Drug Rx:			
None (%)	7 (35)	1 (5.9)	8 (21.6)
ARB (%)	2 (10)	2 (11.8)	4 (10.8)
ACEi (%)	0 (0)	2 (11.8)	2 (5.4)
CCB (%)	1 (5)	1 (5.9)	2 (5.4)
BB (%)	3 (15)	0 (0)	3 (8.1)
AB (%)	0 (0)	1 (5.9)	1 (2.7)
Diuretic (%)	6 (30)	3 (17.6)	9 (24.3)
Statin (%)	2 (10)	1 (5.9)	3 (8.1)
Hydroxychloroquine (%)	0 (0)	12 (70.5)	12 (32.4)
Vitamin D (%)	5 (25)	11 (64.7)	16 (43.2)
MMF (%)	0 (0)	4 (23.5)	4 (10.8)
Monoclonal antibody (%)	0 (0)	4 (23.5)	4 (10.8)
Anti-viral (%)	0 (0)	3 (17.6)	3 (8.1)
General Immunosuppressant (%)	0 (0)	8 (47.1)	8 (21.6)

Systemic lupus erythematosus: SLE. Angiotensin receptor blocker: ARB, Angiotensin-converting enzyme inhibitor: ACEi, Beta Blocker: BB, Alpha Blocker: AB, Mycophenolate mofetil: MMF. All data are expressed as a number of human subjects and percent in parathesis.

**Table 3:** Clinical Characteristics and Demographics of Validation Cohort for Plasma Anti-Endothelin Receptor Autoantibody Biomarkers.

	Non-SLE Non-HTN	Non-SLE HTN	SLE Non-HTN	SLE HTN
N=	45	36	47	49
Age	31 ± 11	48 ± 14	45 ± 14	47 ± 16
Sex (F/M) (% F)	30/15 (66.7%)	29/7 (80.5%)	32/15 (68.1%)	34/15 (69.4%)
Race (W/B/A/O)	(27/17/1/0)	(5/31/0/0)	(11/33/1/2)	(11/37/0/1)
Office SBP (mmHg)	114 ± 9	149 ± 18	126 ± 5	165 ± 15
Office DBP (mmHg)	66 ± 8	82 ± 9	76 ± 7	96 ± 17
Pulse Pressure (mmHg)	48 ± 8	67 ± 19	50 ± 8	68 ± 18

Systemic lupus erythematosus: SLE, Hypertension: HTN, Systolic blood pressure: SBP, Diastolic blood pressure: DBP. All data are expressed as mean ± SD.
